# Exploring resistance and avoidance behaviours at the research delivery, clinical practice interface: group concept mapping through a critical realist lens

**DOI:** 10.1177/17449871241311536

**Published:** 2025-03-19

**Authors:** Linda Tinkler, Steven Robertson, Angela Tod

**Affiliations:** Honorary Research Fellow, School of Allied Health Professions, Nursing and Midwifery, University of Sheffield, Sheffield, UK; Trust Lead – Nursing Midwifery & AHP Research, The Newcastle upon Tyne Hospitals NHS Foundation Trust, Newcastle, UK; Programme Director, RCN Research Alliance, School of Allied Health Professions, Nursing and Midwifery, The University of Sheffield, Sheffield, UK; Professor of Older People and Care, School of Allied Health Professions, Nursing and Midwifery, The University of Sheffield, Sheffield, UK

**Keywords:** communication, health services research, interpersonal leadership, mixed method design, research in practice, teamwork

## Abstract

**Background:**

Clinical research drives health improvement. Perceptions of clinical research by healthcare professionals practising outwith research structures may impact relationships at the research delivery, clinical service, interface and therefore the success of research.

**Aims:**

To establish factors generating resistance/avoidance behaviours displayed by healthcare professionals at the clinical research delivery, clinical service interface.

**Methods:**

Group Concept Mapping from a critical realist perspective was adopted. Participants responded to an open-ended statement, then sorted, rated and interpreted the resulting dataset.

**Results:**

The final concept map contained 99 statements sorted into six conceptual clusters (1) ‘*We value & understand the importance of research*’; (2) ‘*How it should be & how we could work together*’; (3) ‘*Behaviours, beliefs & missed opportunities*’; (4) ‘*Dissonance & disengagement*’; (5) ‘*Time & capacity affects our ability to engage*’ and (6) ‘*I keep thinking of ways to facilitate research as everyone’s business but it is hard*’. Three clusters were rated most likely to generate resistance/avoidance (3, 4 and 5). Two clusters were rated most important to address (2, 5).

**Conclusion:**

This paper contributes previously unheard perspectives on clinical research, indicating several factors generate resistance/avoidance behaviours. Time to engage, opportunities to support studies, improved communication between clinical research and clinical service, and improving awareness from earlier in clinical careers were considered pivotal to success.

## Introduction

The global response to the Coronavirus COVID-19 shifted into focus the value of research activity in facilitating early diagnoses, improving treatments and outcomes, hastening recovery, preventing ill-health, and reducing mortality (Whitehouse et al., 2022). Research was central to the international response to the pandemic including: searching for viable treatment options, (RECOVERY Collaborative Group et al., 2020), developing and deploying vaccination programmes (Lopez et al., 2021), and the generation and analysis of data to inform political strategies on social distancing, facial-mask wearing, and repeated lockdown periods (Scientific Advisory Group for Emergencies, 2020–2022).

The importance of research is evidenced through the associations between healthcare organisation engagement in research and improved healthcare performance and outcomes (Boaz et al., 2015). Evidence also supports the view that research active organisations have lower mortality rates and improved quality ratings (Jonker and Fisher, 2018; Ozdemir et al., 2015).

Delivering research in healthcare systems such as the United Kingdom (UK) National Health Service (NHS) is complicated. Whilst differences in the structure of teams and roles involved in the delivery of research are noted internationally (American Nurses Association, 2016; International Association of Clinical Research Nurses, 2012; Whitehouse and Smith, 2018), a range of challenges can impact on engagement in research. These have the potential to generate tensions at the interface between research delivery and clinical practice in healthcare settings responsible for both agendas (Hernon et al., 2020; McNiven et al., 2021; Tinkler et al., 2018; Tinkler and Robinson, 2020). These tensions negatively affect the success of clinical trials which need to achieve timely recruitment, data collection, and follow-up targets to maximise impact and outcomes.

Research into clinical research recruitment and retention is commonplace. Such activity has led to the development of a dedicated database of recruitment research to support the selection of recruitment strategies for clinical research (Kearney et al., 2018). Additionally, the Trialforge initiative was developed to improve the efficiency of clinical research through increasing the evidence base for decision-making in the design and delivery of studies (Treweek et al., 2015).

The majority of research on research focuses on the practicalities of study delivery, measuring the impact of discrete interventions and their outcomes on improving study efficiency, seeking to achieve marginal gains (Treweek et al., 2018). Optimising recruiter training in relation to equipoise and introducing study participation during treatment discussions have led to a greater awareness of the complexities of study recruitment (Donovan et al., 2016; Mills et al., 2018).

Nationally reported data indicate that in England, research studies consistently struggle to meet recruitment targets (National Institute for Health and Care Research [NIHR], 2022).The Lord O’Shaughnessy review of 2023 highlighted a 44% decline in the initiation of commercial clinical trials in the UK between 2017 and 2021, impacting on global rankings, income generation, and opportunities for patients to benefit from research of relevance to their health (Department and Health and Social Care, 2023a). According to NIHR data presenting activity over the last 6 years, the highest percentage of studies successfully recruiting to time and target in any single quarter was 60.1% (Quarter 2 2019–2020) and the lowest was 51.4% (Quarter 1 2021–2022). Whilst progress has been noted since the government’s response to the Lord O’Shaughnessy review, the highest percentage of National Institute for Health and Social Care Portfolio studies successfully recruiting to time and target in the last reported period of monitoring was 79% (Department of Health and Social Care, 2023a). In an American study of two trial registries containing over 280,000 studies between 1999 and 2020, the majority of studies were reported as small, with the median actual sample sizes in the two databases at 60 and 52. The difference between the target and eventual sample size demonstrated that more studies (on average) failed to hit a target sample size than achieved it (Barnett and Glasziou, 2021). This indicates a significant gap in the efficacy of interventions to improve recruitment to research.

The delivery of clinical research represents one aspect of clinical service delivery; however, evidence suggests it is rarely viewed in this way, and a dichotomy between the two exists (van’t Hoff and Selvaratam, 2018). Subsequently, behaviours displayed towards the Clinical Research Nurse (CRNurse) by colleagues outwith the research team are reported to vary broadly from acting in support of research activity, to displaying gatekeeping behaviours and exhibiting resistance. Gatekeeping behaviours and resistance to research delivery have the potential to jeopardise the success of research and should be addressed (Hernon et al., 2020; Hill, 2018; Tinkler et al., 2018; Tinkler and Robinson, 2020).

## Methodology

### Aims

The aim of this study was to establish and analyse previously unexplored factors, perceived to generate avoidance or resistance behaviours displayed by healthcare professionals who work outside of clinical research structures at the interface between clinical research delivery and clinical service delivery.

The research was undertaken via two phases: a realist review, reported previously (Tinkler et al., 2022), and a group concept mapping study, reported here. The research enabled the exploration of unobservable structures that cause observable events. The events of interest included intentional or unintentional resistance or avoidance behaviours, enacted at the interface between research delivery and clinical service delivery.

The overarching theory below resulting from the realist review (Tinkler et al., 2022) was selected as the foundation for the empirical data collection.

If key colleagues are **not interested in, aware of, or do not understand** the importance, value and utility of research to their role, their patients, or the wider NHS agenda, then they may **unintentionally or intentionally** display avoidance of or resistance to research being delivered in their clinical area. This presents a risk to positive working relationships with CRNurses and access to research opportunities for patients. Consequently, this could impact on organisational performance in relation to research activity and culture.

### Design and theoretical framework

This study used group concept mapping (Kane and Trochim, 2007), working from a critical realist theoretical stance, to collect and then subsequently interpret data to address the study aim. There are six main stages involved in group concept mapping, prior to the final seventh stage where the results are used. These are:

1. Planning
2. Ideas generation
3. Ideas synthesis
4. Organising (a. Sorting and b. Rating)
5. Data analysis (map generation)
6. Interpretation

Group Concept Mapping (GCM) is defined as:

. . . a methodology that creates a stakeholder-authored visual geography of ideas from many communities of interest, combined with specific analysis and data interpretation methods, to produce maps that can then be used to guide planning and evaluation efforts on the issues that matter to the group (Kane and Trochim (2007: 1).

As an integrated mixed methods approach, GCM enables the quantitative analysis of qualitative data, ultimately producing visual representations (maps) of socially constructed theories and ideas about a specific topic, programme or intervention. Researchers have used group concept mapping to understand social and behavioural phenomena in a range of disciplines, with context seen as key to defining the world in which those who are sources of knowledge or opinion operate (Kane and Rosas, 2018).

GCM does not seek to achieve regularity, uniformity or consensus in its approach. Instead, it seeks to illuminate equally, the views of all participants, engaging multiple voices and visually mapping where such voices are similar and different (Kane and Trochim, 2007). This method provides a rich understanding of how different people may view and interpret the same phenomena, from their own contextual position.

Critical realism is defined as a philosophy of the natural and social sciences. It is described as a unique paradigm, positioned on a scale between the extremities of Interpretivism/Constructivism and Positivism (Taylor, 2018). Positivism seeks to objectively identify an independent factual reality that can be applied universally. Interpretivism and Constructivism by contrast focus on interpreting and constructing the discourses, meaning and experiences of people, accepting the existence of a subjective reality (Koopmans and Schiller, 2022). Critical realism combines both structure and interpretation, seeking to achieve a deeper understanding of what is real, what exists, and why. This philosophy aligns with the GCM approach.

### Study setting and recruitment

An invitation to participate in the study was circulated by email to a number of healthcare professional forums to disseminate the opportunity to their members. Opportunities to participate were also posted via the social media platform X (formerly Twitter).

The invitation contained a direct weblink to the Participant Information Leaflet which was held securely on the University webpage. Individuals who opted to participate could follow a direct weblink to the study website, where consent was provided prior to data collection.

The aim was to recruit a minimum of 50 participants to the ideas generation stage. Whilst a range of sample sizes ranging from 10 to 40 at each stage are reported in the literature, this figure has been recommended by Rosas and Kane (2012) to enable sufficient diversity of ideas and adequate richness of data.

It was noted that the target sample of 50 was not achieved and, in comparison to the numbers joining at the outset, relatively low numbers of participants followed through from their chosen initial stage to the next stage of the study and beyond. This was particularly noted in relation to the low number of participants who eventually attended the interpretation session (five expressed intent to attend, three attended on the day). In a pooled analysis of GCM studies, Rosas and Kane (2012) did not assess the validity or impact of interpretation activities, and they stated the intentions of interpretation are to share data with participants and to help participants to understand the data and subsequent interrelationships between. It is not possible to predict the potential impact of a smaller group attending the interpretation session on the overall results of this research. However, the aim of interpretation is to add richness to the recommendations, which was achieved in this study. The discussion and themes emerging during the interpretation session were deemed useful, appropriate, well considered and relevant to the study.

Participant recruitment was opened at ideas generation, then re-opened at sorting and rating to enable additional individuals to join the study at any stage. Table 1 provides information on participant recruitment and flow throughout the study.

**Table 1.** Participant recruitment and flow throughout study.

Ideas generation		Sorting and rating		Interpretation	
Consented	40	Assigned from ideas generation	32	Invited	38
Commenced	38	Commenced sorting	10	Accepted	5
Completed	32	Completed sorting	7	Attended	3
Did not complete	6	Commenced rating	8		
		Completed rating	6		
		Additional recruits consented at sorting/rating	10		
		Commenced sorting	10		
		Completed sorting	3		
		Commenced rating	5		
		Completed rating	3		
**Total contributing ideas**	**32**	**Total completing sorting and rating**	**19**	**Total attending**	**3**

### Ethical considerations

This study involved primary data collection from professionals employed in the NHS. Due to the recruitment approach taken, governance approval from the Health Research Authority was not required. The study received a favourable ethical opinion from The University of Sheffield reference number 037476.

Confidentiality was safeguarded for all participants. The group concept mapping database is hosted in Germany, and privacy policies are such that the hosts have no direct access to participant information or email addresses. Participants were advised not to disclose any identifiable information during data entry, and the general nature of the focus prompt did not risk the disclosure of such data. Investigator checking of the data following ideas generation, confirmed that no identifiable data had been entered. The Data Protection Act (2018) and the UK Policy Framework for Health and Social Care Research (2017) were adhered to throughout this study. Informed consent was received from all participants prior to data collection at all stages of the study.

### Inclusion and exclusion criteria

Eligible participants were Nurses, Nursing Associates, Midwives and the 14 Allied Health Professions defined by NHS England. Participants were not eligible if they were either working within a clinical research delivery role or were leading their own research.

### Data collection and analysis

The six stages of GCM will now be reported including the results at each stage. This approach is necessary to demonstrate how the results of each stage informs the next.

### Stage 1. Planning

Planning includes identifying who will be invited to participate and the development of a statement to enable the collection of relevant views on the topic of interest. This statement is called a ‘focus prompt’ and takes the form of an open-ended sentence which participants complete repeatedly until they have exhausted their views through generating multiple separate statements. Developing the focus prompt is the foundation of collecting the views of participants and needs to be as specific and clear as possible to enable the collection of meaningful data. In the pursuit of upholding a critical realist theoretical position in this research, the focus prompt was designed to reflect the content of the theory taken forward for testing. During planning, four separate, short, focus prompts were developed and tested based on the programme theory selected following the realist review.

The intention was to develop a focus prompt broad enough to elicit a range of authentic views about research delivery without making any inferences that might influence a particular response. Developing a focus prompt that would enable this effectively was a challenge. The focus prompt would need to capture views about the relevance of research to a participant’s role (whether negative or positive) yet be open ended enough to also provide insight into the behaviours present at the interface between clinical research delivery and clinical service delivery.

The following focus prompt, designed to reflect the content of the theory, was used in this study:

A specific view I have about my role in relation to the delivery of clinical research in the NHS is. . .

### Stage 2. Ideas generation

Participation in this stage was undertaken via the Group Wisdom, Concept Systems Global^©^ web-based interface. Consenting participants were asked to respond to the focus prompt, repeatedly completing sentences in order to submit as many responses as they felt able to during this activity. Participants were able to see the anonymous responses of other participants. This is beneficial in developing ideas or building on those of others. The risk of duplication of ideas is also reduced (Kane and Trochim, 2007).

A total of 101 statements were generated by the 32 participants between the 27th of January 2021 and the 30th of April 2021.

### Stage 3. Ideas synthesis

Ideas synthesis removes duplicate statements and ensures the views submitted are clearly articulated (Kane and Trochim, 2007). This stage was led by the first author and checked by two other research team members [AT] and [SR]. The removal of duplicate statements in GCM is a key element, intended to ensure a single set of statements which represents all views submitted. This is beneficial for the subsequent sorting and rating activities providing a condensed dataset, which remains substantial enough to ensure saturation of the themes generated during ideas generation.

Statements not deemed to contain responses relevant to the research aims were removed. Statements containing more than one theme were split into single statements. Statements were also checked for composition, grammar and flow.

The resulting set of 99 unique statements were then shared with participants to enable two further organising activities: sorting and rating.

### Stage 4. Organising

In this stage, participants were invited to complete two activities sequentially: (a) sorting, followed by (b) rating.

#### 4a. Sorting

Participants organised the statements generated in stage one, based on their personal interpretation of how they viewed them in their own context. Sorting involved participants first examining the finalised statement set and then sorting all statements thematically into groups of their choosing using the web-based platform. Participants were free to sort as they saw fit into many or few groups. Participants were asked to label each group of statements with ‘cluster names’. Cluster names provide a thematic title, further insight into how participants had viewed and subsequently sorted the data and are important for analysis (Kane and Rosas, 2018).

#### 4b. Rating

Participants then rated each statement according to two scales. Firstly, based on likelihood of generating avoidance or resistance behaviours at the interface between clinical research delivery and clinical service delivery. Secondly, based on importance to address. Each rating activity was completed using a 4-point rating scale defined in Table 2.

**Table 2.** Rating scales.

Likelihood of generating resistance/avoidance behaviours in relation to clinical research delivery	Importance to address
1 = Not at all likely	1 = Not very important
2 = Somewhat likely	2 = Somewhat important
3 = Likely	3 = Important
4 = Very likely	4 = Very important

At the end of this stage, a complete dataset was quality reviewed, led by the first author and checked by two other research team members [AT] and [SR], prior to being moved into the analysis stage.

## Quality review

Quality review ensures the data are useable in the analysis and match the intentions of the study. Reviewing each participant’s contribution to both sorting and rating is an important step in the research process and links to critical realist philosophy through making judgements about the practical adequacy of each dataset in enabling an accurate and representative analysis (Buch-Hansen and Nielsen, 2020).

Of the ten datasets, nine were confirmed as suitable to take through to analysis. One dataset was rejected because it contained data that had been sorted and labelled categorically rather than conceptually, using categories such as ‘agree’ and ‘disagree’. Due to the fundamental analysis structure for GCM, where data are not sorted conceptually, it may affect the analysis and hamper interpretation if this dataset had been used.

Quality review includes considering to what extent participants reasonably utilise the whole range of the provided rating scale. In this case, a range of between one and four was available on both scales, and participants had used the full range of rating across both scales.

### Stage 5. Data analysis (producing the maps)

The final dataset was used to construct a range of visual representations (maps). Each type of map serves a slightly different purpose and will be explained in the following sections. The analysis and representation were undertaken using the Group Wisdom, Concept Systems Global^©^ online software package. Whilst this software enables several ways in which to represent the data, all visualisations produced are interrelated and are merely different ways of reflecting the views of participants on the same phenomenon (Kane and Trochim, 2007).

**Figure 1.**
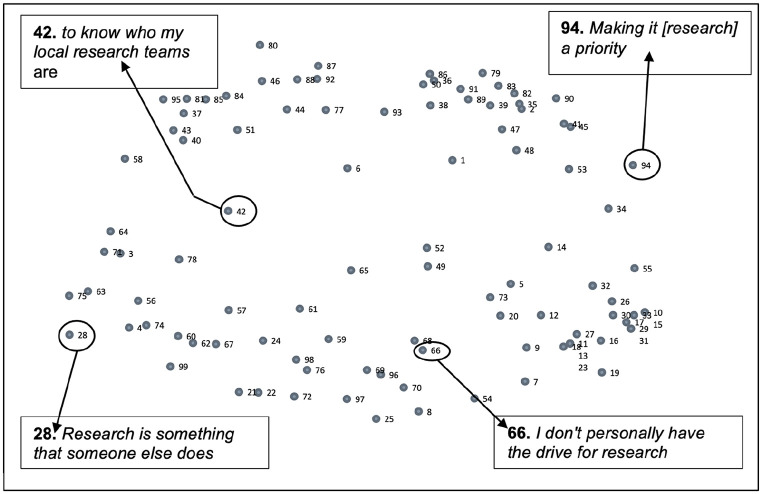
Statement point map.

A similarity matrix is produced first (available via public data file). This is a table of values demonstrating the number of times each statement was sorted together with each of the other statements. The similarity matrix for this study has 99 points on each axis, representing the final 99 statements. The values generated by the similarity matrix were used to create a point map through a process called multidimensional scaling. The statement point map can be seen in Figure 1.

The statement point map represents the data at a statement level as sorted by participants. Each point represents a numbered participant response to the focus prompt. Points are positioned on the map based on how participants sorted them as conceptually similar or disparate to other points during the sorting exercise. Those points closest to each other on the map represent statements that were sorted as closely linked conceptually by participants. Those points further away from each other depict responses that were distinct from each other and therefore less linked.

Following the generation of the point map, to progress the data visualisation, a process called hierarchical cluster analysis produces a cluster map. Remaining true to the positioning of statements on the point map, clusters of statements are generated by grouping the previously sorted points together where they are sorted in closest proximity to each other, representing a set of ideas that together reflect a shared understanding. The cluster map produces a number of coloured shapes based on the proximity of points, and these are populated with the best fit titles to define the themes.

There is no recommended number of clusters; however, it is important to consider the most appropriate number of clusters, in order to reasonably represent the data in sufficient detail with thematic clarity (Kane and Trochim, 2007).

Cluster maps comprising of between 15 and 4 clusters were considered. These were reviewed sequentially, gradually merging clusters together. The assessment of, and arrival at, a preferred cluster solution is a qualitative judgement based on content and fit, remaining true to the sorting activity completed by participants. The goal of gradually merging clusters together is to arrive at a cluster solution which balances the most useful detail between clusters, whilst combining those which appeared practically adequate to merge together, in line with critical realist philosophy (Buch-Hansen and Nielsen, 2020; Kane and Trochim, 2007). The final cluster solution was agreed by the study investigators as six.

**Figure 2.**
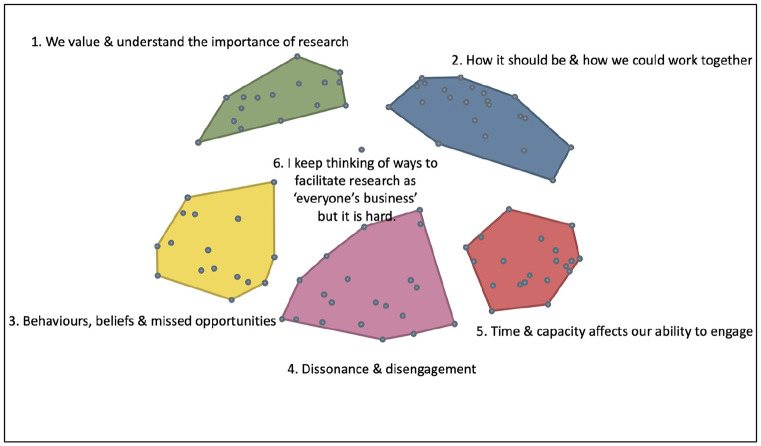
Final cluster solution with amended titles.

Group concept mapping software assigns cluster titles based on those provided by participants for the groups of statements generated in the sorting exercise. By examining the content of statements within a cluster, and their relevance to the cluster title, more meaningful titles were assigned to each cluster. The final cluster map with amended titles can be seen in Figure 2.

Following generation of the final cluster map solution, rating data were overlaid for both rating scales, producing two cluster rating maps. The average participant ratings ranged from 2.13 to 3.20 on a scale between 1 (not at all likely) and 4 (very likely) to generate resistance or avoidance. The average participant ratings ranged from 3.01 to 3.26 on a scale of between 1 (not very important) and 4 (very important) to address.

On average, participants felt that statements contained within clusters three, four and five were more likely to generate resistance or avoidance behaviours, and that statements contained within clusters two and five were most important to address.

**Figure 3.**
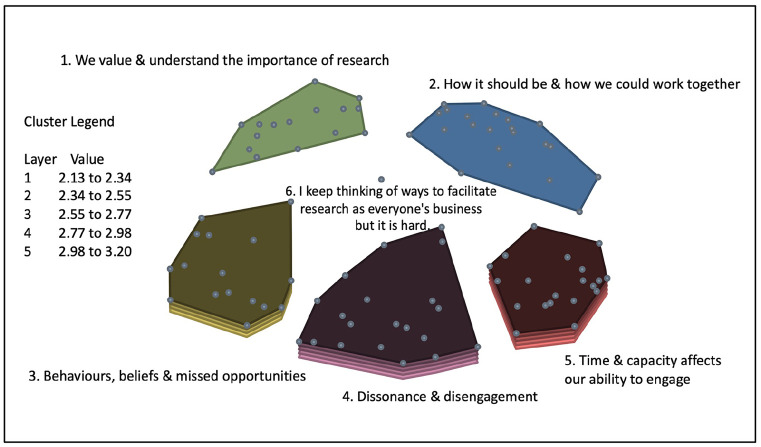
Cluster rating – likelihood of generating resistance or avoidance.

**Figure 4.**
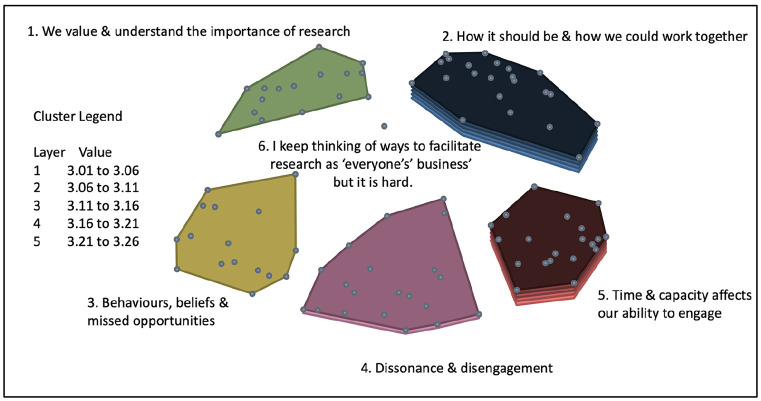
Cluster rating – importance to address.

Figure 3 illustrates the cluster rating map for likelihood of generating resistance or avoidance. Figure 4 illustrates the cluster rating map for importance to address.

Supplemental Table 3 illustrates the full statement set broken down into the six clusters. It also illustrates the average overall rating for each cluster in bold, and then average ratings for each statement across the two rating scales.

### Go zones

**Figure 5.**
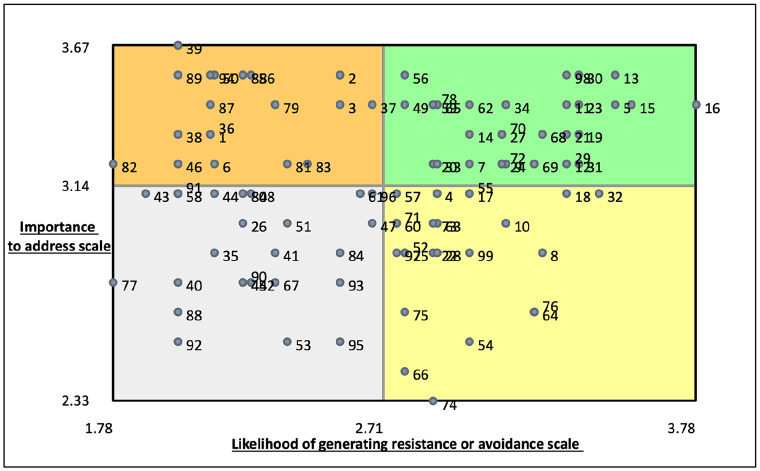
All statements Go zone.

To enable a more detailed analysis of rating data at a statement level, further visualisations called Go zones were produced. A Go zone is a bivariate graph, enabling the assessment of statement level values on both likelihood of resistance or avoidance and importance to address. This is divided into quadrants plotted on an *x-* and *y*-axis, with a vertical line marking the average likelihood rating and a horizontal line marking the average importance rating. This produces a window like visual with the top right quadrant coloured green, identifying those statements rated as both most important to address and most likely to generate resistance or avoidance. The bottom left quadrant colour coded grey represents those statements rated on average as least important and least likely to generate resistance and avoidance. The top left orange quadrant illustrates those statements rated as less likely to generate resistance or avoidance, yet more important to address. The bottom right yellow quadrant contains statements rated on average as more likely to generate resistance or avoidance, but less important to address. Ratings are presented as relative to each other and, in keeping with critical realist philosophy, it is important to acknowledge that this knowledge has been socially produced and is therefore transitive (constantly changing and developing), and subject to limitations. A Go zone, produced for all statements, is illustrated in Figure 5.

Across the whole dataset, statement number 39 was rated as most important to address.

*There should be more opportunities for those not involved in research delivery teams to express an interest in being involved in research projects.*

Statement number 16 was rated as the most likely to generate resistance or avoidance.

*There is limited motivation amongst staff to take an active interest in clinical research delivery because everyone is already overstretched.*

Statement number 74 was rated as least important to address.

*It is up to Doctors to recruit patients*

Statement numbers 77 and 82 were rated as least likely to generate resistance or avoidance.

*The training I have received in order to undertake the clinical interventions for research trials has been beneficial to my clinical role.*

*It’s good for my patients to be able to take part in clinical research when they attend for their care.*

### Stage 6. Interpretation

The final stage is to facilitate participants to interpret the findings using the maps from their perspective. To maximise potential participation, and in light of ongoing variations in lockdowns due to the global pandemic, a participant interpretation session took place via the secure online platform Google Meet^©^. All 38 participants, active at any stage of the study, were invited to attend. Of those invited, five expressed an intention to attend. On the day, three attended and two were unable to attend at short notice due to clinical pressures.

Participants agreed there was evidence of a range of factors and perceptions, likely to generate resistance or avoidance at the interface between clinical research delivery and clinical service delivery. They also expressed the notion that focusing on positive aspects of the data would be an appropriate way forward to improve the success of clinical research delivery.

A number of recommendations were made including a desire to focus on the content of clusters two and five to inform next steps. One participant suggested that, at a statement level, statement number 34. ‘*I lack the support to get as involved in research as I’d like*’. could hold the key to addressing other issues. They further suggested that support to explore, feel comfortable with, and learn about the positive aspects of research should begin at an undergraduate level. The interpretation session enabled a deeper understanding of the collective views and informed the development of a number of recommendations relating to policy, practice and research.

## Discussion

The results of this study suggest healthcare professionals working outside of research continue to have limited understanding of the research delivery landscape and the CRNurse role. This appears to result from a complex range of underlying factors that are inadequately understood. Many statements indicated that participants were cognisant of a range of challenges and complexities within the research landscape. The identification of such challenges may illuminate how the landscape appears to those outwith, and what might be done to support a better understanding of this landscape and integrate it within the wider system.

A number of statements within clusters three, four and five indicated difficulties or discomfort in engaging with research activity and appeared mainly related to the participants’ clinical area or context. These statements indicated a lack of opportunity, time, education or support to engage with research, suggesting that organisational factors may actively prevent engagement. Each of these organisational factors has the potential to be seen as a causal mechanism in generating resistance or avoidance behaviours.

Lack of time and support to engage with research are frequently cited in the international evidence base. This evidence, however, mostly relates to the development of clinical research alongside one’s clinical practice. Consequently, difficulties with carving out research time within a clinical role is one of the most significant barriers reported, and there are frequent calls for protected time to enable a focus on research within clinical practice (Avery et al., 2022; Baltruks and Callaghan, 2018; Caldwell et al., 2017; Trusson et al., 2019; van Oostveen et al., 2017).

Studies have indicated that CRNurses are aware of the limited time they perceive their non-research colleagues may have, to engage with the research they are delivering (Hill, 2018; Tinkler et al., 2018). The results of this study support the view that there is limited time to think about and engage specifically in the delivery of research. Importantly, these views have now been offered by Nurses Midwives and AHPs practising outside of research delivery teams.

A broader understanding of the research landscape is worthy of further consideration in the current context of an increased number of national strategies and plans related to research in the Nursing, Midwifery and Allied Health Professions in England (Department of Health and Social Care, 2021; Health Education England, 2022; NHS England, 2021, 2023; NIHR, 2021, 2022).

England’s Chief Nursing Officer’s strategic plan for research, ‘*Making Research Matter*’ (NHS England, 2021), presents a candid title evoking the view that, historically, research has been considered less important in nursing compared to other professions. This requires bold action to empower and enable nurses to fully embed research within their practice regardless of role.

At the outset, the plan recognises the need for cooperation and non-disruption, acknowledging that close working and engagement will be required to help organisations understand the benefits of giving nurses time to lead, deliver and implement research. The complexities of fostering positive attitudes at all levels, and changing the views of those individuals who do not prioritise research, will be a complex and multifaceted piece of work to achieve in the current healthcare context where a workforce crisis increases the challenge. Global shortages of approximately 6 million nurses, overstretched services, long treatment waiting lists and a reported sub-optimal and bullying culture, all lead to the current low levels of morale and energy reported within the workforce (NHS England, 2019, 2020; West et al., 2020; World Health Organization, 2020).

Some statements highlighted a risk of being bullied for those involved in research, indicating the continued presence of a sub-optimal culture in relation to the clinical research landscape. A number of statements also revealed an explicit lack of personal interest, or a perceived sense of low value and/or importance of research. Such statements demonstrate that there are individuals who are not interested in, not aware of, and do not understand the value of research.

Whilst the results of this study appear to support the view that variations exist in the understanding of research delivery, there is also, in contrast, much positive evidence of awareness, understanding and a sense of value placed in research delivery within clinical settings. Many participants could identify and articulate the importance of research within their clinical role. A number of statements provided evidence of broad acknowledgement of the value and benefits of research being delivered in clinical settings. This correlates with messaging within the NHS Constitution which highlights the obligation placed on healthcare settings to generate and utilise evidence to improve care, whilst also ensuring patients are made aware of opportunities to participate in research relevant to their health (Department of Health, 2015).

Some participants indicated their involvement in, or aspirations to support, research teams with the delivery of their projects. This ranged from ensuring they knew who their local research teams were, to actively supporting and facilitating recruitment and data collection. These views reflect the intentions of the government’s policy paper ‘Saving and Improving Lives, the future of UK Clinical Research Delivery’ (Department of Health and Social Care, 2021). Echoed in the intentions of the Chief Nursing Officer’s plan, this policy paper indicates the government’s ambition is for clinical research to be embedded through a research-positive culture, where all healthcare staff are empowered to support and participate in clinical research as part of their role.

The importance and impact of professional relationships at a range of levels, from understanding the CRNurse role to working cooperatively with the CRNurse, is a key theme apparent across previous literature. Data collected in this study indicate that understanding the work of research delivery teams, and working cooperatively with them, would enhance the broader research landscape.

A notion which has remained present throughout the theoretical reflections in this study was the proposal made by Pawson and Tilley (1997) that the successful implementation of any ‘*programme*’ is reliant on the very minimal requirements of cooperation and non-disruption. According to Pawson and Tilley, the extent to which individuals might either cooperate or disrupt relates directly to the perceived relevance of the particular programme, intervention, policy or strategy to the individual (Pawson, 2006, 2013).

McFadyen and Rankin (2017) highlighted the key role of gatekeepers in enabling research delivery teams to access the patients they are seeking to invite, recruit or follow-up. They acknowledged the complexity of achieving success, suggesting that access to a particular department to deliver research required active cooperation and support rather than simply approval alone. In support of this, Hernon et al. (2020) highlighted the importance of building relationships with colleagues outside of research structures, identifying how ward-based nurses could act as barriers to research study recruitment. This is reflected in the likelihood of generating avoidance and resistance behaviours reported in this study.

The extent to which clinical colleagues outwith clinical research delivery may consciously see themselves as gatekeepers has not been explicitly explored, despite these characterisations being common in the literature. Data generated by this study identify a comparative frustration and a sense of being an outsider on *their* part, as they express a wish to know more about research activity in their clinical area and a desire for better communication between themselves and CRNurses. Their articulation of such aspirations includes, in some cases, placing responsibility for improved communication with the CRNurse and research delivery team. In contrast, some participants acknowledged their own responsibility to be informed and to seek out CRNurses in order to offer their support with identifying patients for studies.

These opposing narratives provide some insight into the variety of views that emerged regarding communication between clinical research delivery structures and clinical service delivery. An understanding of the extent to which individuals feel empowered and able to influence a given scenario, or indeed whose responsibility they feel it is, could be a significant mechanism in generating resistance or avoidance behaviours at the interface between clinical research delivery and clinical service delivery. These contrasting views may be partially explained by the concept of individual locus of control, a psychological, social learning theory, developed by Julian Rotter in 1966. The concept describes the extent to which an individual has perceived control over their life and environment. It is a core element in understanding how people live in and interact with their world (April et al., 2012).

Whilst individual efforts may contribute to improving relationships in a specific context, neither an increased commitment nor effectiveness in communication on the part of the CRNurse, nor indeed the efforts of the individual outwith research, can address such systemwide challenges alone. Rather, at organisational and national levels, the reality of time and capacity constraints affecting the ability to engage remains a key generator of behaviours that inevitably affect the success of studies.

In support of this, at a statement level, statement number 16 ‘*There is limited motivation amongst staff to take an active interest in clinical research delivery because everyone is already overstretched*’ was rated as the single statement most likely to generate resistance and avoidance behaviours across the whole study.

In contrast, statement number 39 ‘*There should be more opportunities for those not involved in research delivery teams to express an interest in being involved in research projects.*’ reflects a positive appetite towards involvement and engagement in research. These two extremes provide insight into variations in culture and staff experience evident across the NHS. Furthermore, they serve to confirm the relevance of a critical realist perspective in acknowledging the importance of context and in considering the potential utility and practical adequacy of interventions.

West et al. (2020) acknowledged the unprecedented pressure the COVID-19 pandemic had placed on the UK health service. However, they also noted that many of these pressures were not new, and the pandemic served only to exacerbate pressures already significantly impacting on nurses and midwives. Inequalities, unprecedented and unrelenting pressures at work, and sub-optimal working conditions had already led to an increasing gap between capacity and demand in relation to vacancy rates. The Courage of Compassion paper (West et al., 2020), which sets out eight recommendations in relation to supporting nurses and midwives to practise in psychologically safe and supported workplaces, indicated that the nursing and midwifery workforce requires three core needs to be met to tackle stress and to improve motivation at work. These three needs are neither financially costly, nor unrealistic; they are autonomy, belonging, and contribution.

The concept of autonomy relates to assumed constraints. An assumed constraint is a perceived limitation of experiences, both current and in the future based on experiences of the past. They are some of the most common and powerful constraints in the workplace and may develop over time as resilience is reduced and repeated experiences result from negative reinforcement (Blanchard, 2010).

Combining the range of global evidence with this study’s results, it is reasonable to suggest that for research to succeed, CRNurses too, will require autonomy and belonging. They should expect to be developed, feel valued, respected and supported for their contribution, regardless of where in the patient pathway that contribution is made. To achieve this, research activity will need to be fully established as a golden thread, weaved into the daily lives of every health and social care professional in practice, and much work will be required to improve communication at the research practice interface.

## Strengths and limitations of the work

The utility of exploring issues through a Critical Realist lens enabled views to be collected that took account of the inherent complexity and social nature of research delivery structures within the NHS, and how they interfaced with other clinical service structures.

The web-based GCM platform enabled the continuation of the research during a time when face-to-face data collection was not feasible (COVID-19). Participants were able to access the study remotely, and at their convenience, within the time-constraints of each of the phases.

The challenges related to participant numbers and continuation across the different steps in the empirical data collection meant that effective subgroup analysis was not possible. It also became apparent, after the completion of ideas generation, sorting and rating activities, that one of the participant demographics questions had failed in the web-based platform. The area of practice was not populated by any participant, despite it being compulsory. It was therefore not possible to extrapolate any inferences from this data. Furthermore, due to low numbers participating beyond the original ideas generation step, it was not possible to draw any inferences regarding subgroup views on the likelihood of generating resistance or avoidance, and on those issues seen as important to address.

It was noted that in comparison to the numbers joining at the outset, relatively low numbers of participants followed through from their chosen initial stage to the next stage of the study and beyond. This was particularly noted in relation to the low number of participants who eventually attended the interpretation session (five expressed intent to attend; however, three attended on the day). Whilst there is a risk of criticism in relation to the low attendance at the interpretation session, in a pooled analysis of GCM studies, Rosas and Kane (2012) did not assess the validity or impact of interpretation activities. The intentions of interpretation are to share data with participants and to help participants to understand the data and subsequent interrelationships. It is not possible to predict the potential impact of a smaller group attending the interpretation session on the overall results of this research; however, the aim of interpretation is to add richness to the recommendations, which was achieved in this study.

The web-based nature of this study generated some limitations which might have been avoided with a face-to-face approach. These included participants sorting statements in a binary manner rather than thematically, and not completing all aspects of the processes (sorting and rating) despite reminders. Challenges, related to participant numbers and continuation across the different stages, meant that subgroup analysis was not possible.

Recruitment was impacted by the COVID-19 pandemic. While the study was retweeted 586 times and liked 493 times, these numbers did not translate into the same number of individuals participating in the study.

Finally, as with many studies, participants were self-selecting, and this could mean that only those with strong views on the interface between clinical service and clinical research delivery were motivated to take part. However, the statements produced during ideas generation (step 2) suggest a broad range of views were captured.

## Implications and recommendations

The implications of this study highlight the continuing need to better integrate research activity within healthcare settings. The following recommendations are offered:

1. Work should be undertaken to address where negative behaviours are displayed at the interface between clinical research delivery and clinical service delivery, and to improve communication and team working. This should include work to facilitate staff in understanding the impact of their perceptions and resulting behaviours on both those who work alongside and the patients who may subsequently miss opportunities to participate in research.
2. Consideration should be given to how current healthcare system hierarchies and structures act as mechanisms in generating resistance and avoidance behaviours. Work should be undertaken to map the recommendations from West et al. (2020) directly to the interface between clinical research delivery and clinical service delivery.
3. Undergraduate research awareness programmes and placements should be introduced widely as a priority within the main undergraduate curriculum for Nursing, Midwifery and Allied Health professions. Whilst this may present challenges to placement capacity and require bold thinking, it will be critical to future success and should be incorporated into future policy work. The NIHR has already begun to address this through the INSIGHT Programme.
4. Future approaches to the implementation of the CRNurse role should learn from, and take greater account of, previous research related to flexible team structures and embeddedness.
5. Targeted training and development, with the aim of increasing autonomy and knowledge to get involved with the delivery of research, should be designed and implemented at a range of levels across the healthcare system. This work should continue until research becomes fully embedded as part of the fabric of clinical service provision, and leaders at all levels are able to articulate the potential and value of research occurring in their area, and for them to be empowered to support this.

## Conclusions

This study contributes unique insights into the perceptions and experiences of healthcare professionals practising outwith research delivery structures on the clinical service delivery/clinical research interface. These perspectives confirm the existence of sub-optimal attitudes and perceptions with the potential to generate resistance or avoidance behaviours in some contexts. This work has also illuminated previously unheard perspectives, indicating that research delivery is valued, understood and supported in some contexts. Such views may challenge aspects of the current evidence base related to sense of value and provide further encouragement in relation to what is needed to address the remaining issues.

Eliciting the views of healthcare professionals practising outwith research delivery structures on what could and should be done differently to improve the success of clinical research delivery provides an opportunity for their perspectives to be heard and considered. The main aspects highlighted relate to a desire for time and support to engage with research to be enabled within clinical practice. Support and encouragement to engage with research should commence at undergraduate levels, but should continue to be supported at all levels systemwide. Improved communication between clinical research delivery teams and clinical service delivery will be pivotal to the success of research and a reduction of resistance and avoidance behaviours. The extent to which addressing these aspects might improve access to research for patients and the success of research delivery is not yet known and has been recommended as worthy of further exploration.

Those leading and setting the priorities for the NHS espouse a commitment to research, as identified in a range of strategies, plans and policies discussed in this paper. The extent to which these are supported and enabled in subsequent years depends on the cooperation and non-disruption of the workforce at a range of levels. This study has highlighted a number of key issues related specifically to relationships at the interface between clinical research delivery and clinical service delivery. The extent to which these issues will continue to generate mechanisms such as avoidance and resistance behaviours depends on the successful enabling of less tangible constructs debated across leadership literature, such as locus of control, autonomy, psychological safety, and culture.

Whilst conducted in the English NHS, the results of this study may be transferrable to other countries and contexts. The methods reported here may provide a way forward for other countries to explore their own contexts and may provide an alternative foundation upon which to investigate the interpersonal aspects vital to the successful delivery of research. In settings where opportunities to engage with research delivery are few, then interest in or understanding of research will be reduced. It is reasonable to conclude that where a lack of interest, awareness or sense of value in a particular subject exists, then behaviours that project a sense of reluctance to engage, or a sense of frustration at not feeling more informed or involved, will likely follow.

**Key points for policy, practice and/or research**

- This study confirms the existence of factors likely to generate resistance or avoidance behaviours at the interface between clinical research delivery and clinical service delivery, and identifies what could work better at this interface. This has importantly been articulated by those practising outwith research structures, providing a unique contribution to the evidence base.
- This study provides new evidence that healthcare professionals practising outside of clinical research structures would like opportunities to be involved in supporting research delivery, yet are often prevented by the structures in which they work.
- Courageous thinking is required to enable healthcare professionals positioned either side of the perceived clinical research delivery/clinical service delivery interface to increase research awareness, involvement, and support.
- Improved communication between clinical research delivery teams and clinical service delivery is pivotal to ensuring both access to research for patients and the success of research. To enable this, there is a need to formally challenge hierarchies, structures and workforce configuration to reduce tension at the interface between these important roles and enable closer/integrated working for the benefit of the wider population.

## Supplemental Material

sj-pdf-1-jrn-10.1177_17449871241311536 – Supplemental material for Exploring resistance and avoidance behaviours at the research delivery, clinical practice interface: group concept mapping through a critical realist lens Supplemental material, sj-pdf-1-jrn-10.1177_17449871241311536 for Exploring resistance and avoidance behaviours at the research delivery, clinical practice interface: group concept mapping through a critical realist lens by Linda Tinkler, Steven Robertson and Angela Tod in Journal of Research in Nursing
