# Supplementary material for: Exploring resistance and avoidance behaviours at the research delivery, clinical practice interface: group concept mapping through a critical realist lens
Source: J Res Nurs. 2025 Mar 19;30(3):233–53. doi: 10.1177/17449871241311536 (PMC11924056; doi:10.1177/17449871241311536)
Supplement: sj-pdf-1-jrn-10.1177_17449871241311536 – Supplemental material for Exploring resistance and avoidance behaviours at the research delivery, clinical practice interface: group concept mapping through a critical realist lens [file sj-pdf-1-jrn-10.1177_17449871241311536.pdf]

Cluster Title	STATEMENTS		Average Likelihood of resistance or avoidance	Average Importance
**1. We value and understand the importance of research**			**2.18**	**3.03**
	37	The importance of promoting research engagement to other nurses and AHP's	2.67	3.44
	40	My role supports research in practice	2	2.78
	43	To ensure my local research teams know me	1.89	3.11
	44	To ensure my local research teams know how I can help	2.13	3.11
	46	To be aware of ongoing research projects	2	3.22
	51	To play a role in helping the recruitment process to happen.	2.38	3
	58	Research should be made more visible and exciting.	2	3.11
	77	The training I have received in order to undertake the clinical interventions for research trials has been beneficial to my clinical role	1.78	2.78
	80	Research is the key part of how we improve the lives of people with an LD	2.22	3.11
	81	Research is the key part of how we improve LD services and keep moving forward	2.38	3.22
	84	I understand the importance of research	2.56	2.89
	85	That everyone who has clinical contact should have a basic understanding of the principles of clinical research	2.22	3.56
	87	Research should be at the core of what we do as nurses	2.11	3.44
	88	My role is pivotal to improve research on the front line	2	2.67
	92	I think it would be really interesting	2	2.56
	95	Research in large teaching hospitals is given a lot of credibility	2.56	2.56
**1. How it should be and how we could work together**			**2.26**	**3.21**
	34	I lack the support to get as involved in research as I'd like.	3.13	3.44
	47	To help with identifying appropriate patients if they fit study criteria.	2.67	3
	2	Increased collaboration between clinical staff and research staff	2.56	3.56
	93	Being proactive	2.56	2.78
	83	Clinical research is important to the NHS	2.44	3.22
	53	The more 'customers' we recruit, the higher the chances of retaining funding	2.38	2.56
	41	To help researchers with their projects	2.33	2.89
	79	To enable better care for our patients	2.33	3.44

	48	It could be part of the routine nurses work to seek out appropriate trials and or patients	2.25	3.11
	86	It should be integral to my clinical work.	2.25	3.56
	45	To facilitate data collection for research if approached by members of the research team and asked to help with this.	2.22	2.78
	90	It is encouraged	2.22	2.78
	35	As a clinician, I am perfectly placed to work alongside the delivery team. I screen, recruit, take consent and deliver interventions etc with the support of my delivery team	2.13	2.89
	50	It is important to be involved or advise our patients about clinical research as it improves patient care.	2.13	3.56
	1	Effective team working is essential between clinical and research delivery teams	2.11	3.33
	36	That everyone has a responsibility to enable clinical research to take place in the NHS	2.11	3.33
	94	Making it a priority	2.11	3.56
	38	I believe I have a duty of care to at least participate in research as a clinician.	2	3.33
	39	There should be more opportunities for those not involved in research delivery teams to express an interest in being involved in research projects.	2	3.67
	89	Nurses should be supported to be involved in research at all levels of their clinical roles.	2	3.56
	91	It provides an opportunity to work collaboratively with people you wouldn't usually work closely with	2	3.11
	82	It's good for my patients to be able to take part in clinical research when they attend for their care	1.78	3.22
**2. Behaviours, Beliefs & Missed Opportunities**			**2.79**	**3.01**
	3	There are frequently silos of research going on which would and could influence care delivery and efficiency if there was adequate communication channels	2.56	3.44
	4	People who do research or bring innovation are likely to be bullied	2.89	3.11
	28	Research is something that someone else does	2.89	2.89
	42	To know who my local research teams are	2.25	2.78
	56	Research studies need to explain their purpose in a way that junior and unregistered nurses can engage with.	2.78	3.56
	60	I find research dull and difficult to understand	2.75	3
	62	Research is invisible to nurses	3	3.44
	63	Research is invisible to patients	2.89	3
	64	I have no idea what research studies are currently being recruited for	3.22	2.67
	67	I do not really have anything to do with it	2.33	2.78
	71	Unaware of any relation of my role to clinical research in the nhs in my current trust	2.75	3

	74	It is up to Doctors to recruit patients	2.88	2.33
	75	Do what you are told to do, and support the doctors with their research involvement.	2.78	2.67
	78	There is a historical belief that clinical research nurse roles can't be undertaken by non nurses (ie AHPs). I think this should be encouraged to change	2.88	3.44
	99	The majority of people I know leave clinical roles to work in research	3	2.89
	57	Junior nurses do not have enough knowledge of ongoing research and the importance of it.	2.75	3.11
**3. Dissonance and disengagement**			**2.99**	**3.09**
	8	Usually laborious and requires heavy admin work	3.25	2.89
	25	Research is an add on	2.78	2.89
	54	Research has become more about money and less about patients	3	2.56
	66	I don't personally have the drive for research	2.78	2.44
	68	Research is seen as elitist	3.25	3.33
	69	I don't find out the outcome of the research I support	3.22	3.22
	70	There is limited motivation amongst staff to take an active interest in clinical research delivery because they don't feel the results will have a direct impact on their work.	3.11	3.33
	96	Lots of community staff believe that research is a highly technical, advanced activity	2.67	3.11
	97	Lots of community staff believe that research happens in hospital	2.75	2.89
	49	There are frequently missed opportunities for district nursing staff to identify and signpost potential research participants toward appropriate studies.	2.78	3.44
	52	We were asked to treat research as a 'business'.	2.78	2.89
	65	Nurses should know about research in their area, but we don't	2.89	3.44
	61	I am not very well informed about research	2.63	3.11
	76	The clinical trials associated with the department I work for and the university we are affiliated to have limited focus on non-pharmacological clinical research	3.22	2.67
	59	There is lot of misunderstanding about clinical research in NHS	2.88	3.44
	98	Lots of community staff believe that research doesn't apply to them	3.33	3.56
	72	There is inconsistency of approach	3.11	3.22
	24	Clinical care and research are seen as separate entities	3.11	3.22
	22	Research is not seen as something to make an effort to engage with in Learning Disability services	2.88	2.89

	21	Research is not seen as necessary in Learning Disability services	3.33	3.33
**5. Time and capacity affects our ability to engage**			**3.2**	**3.26**
	5	People who do research or bring innovation lack peer support	3.5	3.44
	7	I have been involved in the delivering the clinical intervention of two different research trials but this has been alongside my own clinical caseload and has caused increased pressure	3	3.22
	9	It sometimes can be time consuming	3.13	3.22
	10	I always try to engage and support research activities in the unit but sometimes time limits my capacity	3.13	3
	11	It is hard to be involved within our working hours.	3.33	3.44
	12	I lack the time to get as involved in research as I'd like	3.33	3.22
	13	Time isn't prioritised within clinical working to allow engagement in research	3.5	3.56
	14	Education isn't prioritised within clinical working to allow engagement in research	3	3.33
	15	We just don't have the time	3.56	3.44
	16	There is limited motivation amongst staff to take an active interest in clinical research delivery because everyone is already overstretched	3.78	3.44
	17	The delivery of research requires further learning away from patient care	3	3.11
	18	The delivery of research requires further time away from patient care	3.33	3.11
	19	Research it is often hard to integrate it into my clinical practice as it is extra to my workload.	3.38	3.33
	20	Research is not taken into consideration on a day to day basis in a clinical setting	2.88	3.22
	23	Because research is seen as 'an add on' instead of part of clinical care, clinicians don't have time to engage in research.	3.38	3.44
	26	Research is a nice extra	2.22	3
	27	Research is seen as a bother rather than core business	3.11	3.33
	29	Often clinical research is not seen as a priority within the department	3.33	3.22
	30	It is not often considered a priority by managers or heads of services.	3.38	3.56
	31	Clinical research is expected to take a back seat in order for clinical practice to be delivered	3.38	3.22
	32	Patient contact is seen to be of more importance than research in a clinical setting	3.44	3.11
	33	I always try to engage and support research activities in the unit but sometimes the need to prioritise patient care limits my capacity	2.89	3.22
	55	The challenges of embedding clinical research in the NHS during various pressures (money,	3	3.11

		workforce, pandemic etc.) is costing patients opportunities of accessing clinical trials.		
	73	The clinical trials associated with the department I work for and the university we are affiliated to are very medically focused	2.88	3
**6. I keep thinking of ways to facilitate research as 'everyones' business' but it is hard.**			**2.13**	**3.22**
